# Involuntary Memories after a Positive Film Are Dampened by a Visuospatial Task: Unhelpful in Depression but Helpful in Mania?

**DOI:** 10.1002/cpp.1800

**Published:** 2012-05-09

**Authors:** Davies Charlotte, Aiysha Malik, Arnaud Pictet, Simon E Blackwell, Emily A Holmes

**Affiliations:** Department of Psychiatry, University of OxfordOxford, UK

**Keywords:** Mental Imagery, Positive Emotion, Intrusive Memory, Involuntary Memory, Mania, Tetris

## Abstract

Spontaneous negative mental images have been extensively researched due to the crucial role they play in conditions such as post-traumatic stress disorder. However, people can also experience spontaneous positive mental images, and these are little understood. Positive images may play a role in promoting healthy positive mood and may be lacking in conditions such as depression. However, they may also occur in problematic states of elevated mood, such as in bipolar disorder. Can we apply an understanding of spontaneous imagery gained by the study of spontaneous negative images to spontaneous positive images? In an analogue of the trauma film studies, 69 volunteers viewed an explicitly positive (rather than traumatic) film. Participants were randomly allocated post-film either to perform a visuospatial task (the computer game ‘Tetris’) or to a no-task control condition. Viewing the film enhanced positive mood and immediately post-film increased goal setting on a questionnaire measure. The film was successful in generating involuntary memories of specific scenes over the following week. As predicted, compared with the control condition, participants in the visuospatial task condition reported significantly fewer involuntary memories from the film in a diary over the subsequent week. Furthermore, scores on a recognition memory test at 1 week indicated an impairment in voluntary recall of the film in the visuospatial task condition. Clinical implications regarding the modulation of positive imagery after a positive emotional experience are discussed. Generally, boosting positive imagery may be a useful strategy for the recovery of depressed mood. Copyright © 2012 John Wiley & Sons, Ltd.

People can experience positive imagery that springs to mind unexpectedly. In a classic example from literature, Marcel Proust (1913/2001) wrote that

No sooner had the warm liquid mixed with the crumbs touched my palate than a shudder ran through me and I stopped, intent upon the extraordinary thing that was happening to me. An exquisite pleasure had invaded my senses…Whence could it have come to me, this all-powerful joy?… Undoubtedly what is thus palpitating in the depths of my being must be the image, the visual memory which, being linked to that taste, is trying to follow it into my conscious mind. (Proust, 1913/2001, pp. 45–46)

This example from Proust illustrates the sudden occurrence of a spontaneous involuntary emotional memory. Spontaneous negative images have been well researched in the context of flashbacks—image-based memories that are the hallmark characteristic of post-traumatic stress disorder (PTSD; American Psychiatric Association, 2000). Such intrusions play a critical role in the maintenance of PTSD and offer a treatment target (Brewin & Holmes, 2003; Ehlers & Clark, 2000). There is increasing evidence that such spontaneous negative images not only are important in PTSD but also contribute to the distress experienced in other emotional disorders such as social phobia (Hirsch, Clark, Mathews, & Williams, 2003), agoraphobia (Day, Holmes, & Hackman, 2004), depression (Patel et al., 2007) and so forth. There has been a growing body of research aimed at understanding the role of spontaneous negative imagery in emotional disorders (Brewin, Gregory, Lipton, & Burgess, 2010; Holmes & Mathews, 2010).

People can also experience spontaneous positive mental images, as the excerpt from Proust illustrates. However, there is a relative lack of research concerning these. What might be the impact of experiencing such spontaneous positive images? We shall focus on two possible aspects: (1) positive imagery may fuel positive mood and protect against depressed mood and (2) excessively positive imagery may fuel mania and potentially destructive drives.

First, positive imagery may play a role in promoting positive mood. It has been suggested that a focus on positive cognition and emotion (rather than merely negative cognition) provides a fruitful avenue for treatment development (MacLeod & Moore, 2000). Spontaneous positive imagery makes up a significant part of day-to-day experience. For example, in the general population, approximately half of all involuntary memories may be positive (Berntsen, 1996; Berntsen & Jacobsen, 2008), and in a healthy sample, spontaneous future-oriented thoughts were reported as more frequently positive than negative (D'Argembeau, Renaud, & Van der Linden, 2011).

Conversely, depression is associated with deficits in positive cognition about both the future and the past. For example, in a future fluency paradigm, depressed individuals generated fewer positive future positive events and had reduced expectancy of enjoying them compared with non-depressed participants (MacLeod & Byrne, 1996; MacLeod & Conway, 2007). On a task requiring deliberate generation of future imagery, Holmes, Lang, Mould, and Steel (2008) found that low mood was associated with reduced vividness of positive future imagery amongst healthy participants. In a subsequent clinical study, participants with major depressive disorder generated less vivid future imagery than a healthy control group (Morina, Deeprose, Holmes, Pusowski, & Schmid, 2011). In relation to past cognition, formerly depressed individuals recall less vivid positive memories following a negative mood induction (Werner-Seidler & Moulds, 2011), and when recovered or currently depressed individuals do recall positive memories, this may lead to mood deterioration rather than improvement (Joormann, Siemer, & Gotlib, 2007). Given these deficits in effortful positive cognition in depression, enhancing positive cognition, e.g., by promoting positive imagery, may offer an exciting treatment development target (Blackwell & Holmes, 2010; Lang, Blackwell, Harmer, Davison, & Holmes, 2012).

The mechanisms underlying the deficit in positive cognition observed in depression have been investigated with regards to a number of different hypotheses, of which we will summarize a brief selection. In relation to memory, difficulty in accessing positive memories has been understood in terms of the differential activation hypothesis (Teasdale, 1988; Werner-Seidler & Moulds, 2011), and there is some evidence that a ruminative, verbal style of processing may reduce the potential beneficial impact of recalling positive memories (Werner-Seidler & Moulds, in press). In relation to future cognition, the deficits observed in depression have been related to difficulty in accessing positive autobiographical information, e.g., due to deficits in positive affect (MacLeod, Tata, Kentish, & Jacobsen, 1997) or overgeneral memory (Sarkohi, Bjärehed, & Andersson, 2011; Williams et al., 1996). Depressed individuals do seem able to access positive future events on a semantic level but struggle to engage with them as personal possibilities, perhaps due to reduced estimates of the likelihood of such events occurring to oneself (MacLeod & Conway, 2007).

Second, there is emerging evidence that spontaneous ‘positive’ images can also occur in problematic states of elevated mood, such as in bipolar disorder (Deeprose, Malik, & Holmes, 2011; Gregory, Brewin, Mansell, & Donaldson, 2010; Holmes et al., 2011). It is hypothesized that such positive imagery can amplify mood and drive manic behaviour (Holmes, Geddes, Colom, & Goodwin, 2008), e.g., via images of goals (e.g., of driving a racing car). Risk for mania has been associated with highly ambitious goal setting and increased achievement-related goals (Gruber & Johnson, 2009; Johnson, 2005; Johnson & Carver, 2006). Other potentially destructive conditions have recently been associated with imagery that is positive/appetitive at the time, such as suicidal imagery (Crane, Shah, Barnhofer, & Holmes, 2012; Hales, Deeprose, Goodwin, & Holmes, 2011; Holmes, Crane, Fennell, & Williams, 2007) and substance-misuse craving (May, Andrade, Panabokke, & Kavanagh, 2004). Thus, although promoting spontaneous positive imagery may be a desirable treatment target in depression, in mania, dampening down such imagery may be more helpful.

To summarize, positive spontaneous images seem to be a key part of normal day-to-day experience but may be problematic in their absence (as in depression) or excessive proliferation (as in hypomania/mania). Although it is hypothesized that the potentially deleterious effects of imagery in hypomania/mania are in amplifying positive mood and via their impact on goal-directed behaviour (Holmes, Geddes, et al., 2008), it is not yet known what aspect of the experience of positive spontaneous images might be involved in this process. Possible candidates might be number or frequency of images, appraisals of the images, qualities of the images or some combination. Developing a paradigm to experimentally investigate the formation and the manipulation of spontaneous positive images would therefore aid investigation of this phenomenon and enable us to explore how an experience that may be beneficial, yet lacking, in depression may even become unhelpful in mania. It should also reveal how we might influence the formation and the experience of such images via an imagery-interfering task.

An experimental paradigm to investigate the formation of spontaneous images has already been developed, albeit in the area of trauma research with negative intrusive memories as the focus. In the context of trauma, intrusive memories can range from fleeting sensory impressions of the traumatic events to ‘full-blown’ flashbacks that are so intense the individual dissociates and feels as though he or she is back at the time of the trauma. However, the latter are very rare, and the former, much more common, intrusive memories are clinically important in the maintenance of PTSD (Ehlers & Clark, 2000; Foa, Hembree, & Rothbaum, 2007). We use the term ‘flashback’ to describe vivid sensory-perceptual (predominantly visual images) emotional memories from an event that seems to intrude involuntarily into consciousness (Holmes, James, Coode-Bate, & Deeprose, 2009; Holmes, James, Kilford, & Deeprose, 2010).

Our understanding of the development of spontaneous negative imagery such as flashbacks has been developed using the trauma film paradigm (Holmes & Bourne, 2008). In this paradigm, participants watch a film depicting traumatic scenes of injury and death. Over a subsequent period, participants typically experience spontaneous intrusive images, or ‘flashbacks’, from the film, which are recorded in a diary. It was predicted that tasks that interfere with the ability to consolidate visual memories (visuospatial tasks) would selectively impair the development of intrusive visual images. This prediction was derived from both experimental psychology and clinical theories that memory differentiates visual and verbal components (Paivio, 2007). In relation to trauma memory, flashbacks consist of sensory, visual images (Ehlers, Hackmann, & Michael, 2004). Visuospatial cognitive tasks compete for processing resources with visual images (Holmes, Brewin, & Hennessy, 2004) and may therefore interfere with memory consolidation of these flashbacks by competition for the same limited cognitive resources. Consistent with this theory, in a series of studies, flashback development was found to be reduced by visuospatial (but not certain verbal) tasks during or shortly after the film (Bourne, Frasquilho, Roth, & Holmes, 2010; Deeprose, Zhang, DeJong, Dalgleish, & Holmes, 2012; Holmes, James, et al., 2009; Holmes et al., 2004; Holmes et al., 2010). This is not simply an effect of distraction, as other types of tasks such as verbal tasks have been demonstrated to lead to increased flashbacks when engaged in during or after viewing of a trauma film (Holmes & Bourne, 2008; Holmes et al., 2010). Two recent papers used a widely available visuospatial task comprising the type of game played widely in daily life—the computer game ‘Tetris’ (Tetris Zone, 2007). As the differentiation of verbal and visual components is thought to be a general feature of memory, rather than limited to negative memories, we might expect the effects described above to apply to positive as well as negative memories. However, little work has addressed this to date.

Recent work has successfully translated one experimental paradigm used to investigate negative memories to the study of deliberately recalled positive memories, investigating techniques for dampening deliberately recalled negative memories in the context of positive memories (Engelhard, van Uijen, & van den Hout, 2010; van den Hout et al., 2010). In this paradigm, participants were required to form images of memories of three negative and three positive occasions. While recalling the memories, participants were asked to make horizontal eye movements, play Tetris or keep their eyes stationary (recall only). Either making eye movements or playing Tetris decreased emotionality ratings and physiological startle responses compared with recall only. Although there was a broadly similar pattern of results for both positive and negative memories, there were some differential effects of the two tasks that highlight the need for further investigation in this area (Engelhard et al., 2010). Further, this study examined *deliberate* recall of memories, and it therefore remained to be tested whether we can translate a different paradigm used to study the development of negative *involuntary* memories to the study of positive *involuntary* memories. In particular, although a visuospatial task impedes the development of *negative* involuntary memories (e.g., Holmes et al., 2010), it was untested whether the same task reduces *positive* involuntary memories. By extension of the theory of the development of involuntary negative memories, we might expect that a visuospatial task would also interfere with the consolidation of sensory, image-based memories of positive emotional material, leading to fewer involuntary memories.

The current study extended the trauma film paradigm by developing an overtly ‘positive’ film, drawing on themes associated with mania. We predicted that participants would show higher scores on measures of positive affect and goal setting immediately after watching the film. Further, we predicted that participants would report positive involuntary memories of the film over the next week. Finally, we predicted that compared with those engaging in no task post-film, those participants engaging in the visuospatial task ‘Tetris’ would report fewer involuntary memories of the positive film over the subsequent week.

## METHOD

### Participants

Ethical approval was obtained from the University of Oxford Central University Research Ethics Committee, reference MSD/IDREC/C1/2010/82. Volunteers (39 women, 32 men, *M*_age_ = 20.3 years, *SD* = .87, age range = 18–22 years) were recruited via advertisements in university premises. Participants were entered into a prize draw of £50 and received chocolate following participation in the study. Seventy-one participants were recruited, and two participants (no-task condition) did not return intrusion diaries and were excluded from analyses. Data are therefore presented for the 69 participants who returned intrusion diaries.

### Materials

#### Positive film

The 15-min 15-s film contained seven clips selected to contain themes deemed in some way related to diagnostic criteria for mania, such as elevated or expansive mood, inflated self-esteem or grandiosity, heightened energy, decreased need for sleep, goal attainment and thrill seeking (American Psychiatric Association, 2000; Gruber & Johnson, 2009). The film focused on themes related to ‘positive’ aspects of the experience of mania and thus did not include themes related to irritability, which is also a diagnostic feature. As the participants were students in Oxford, the selection of clips was tailored to be personally relevant to this population (Gruber, Johnson, Oveis, & Keltner, 2008). Content of clips included a first-person point-of-view rollercoaster ride at Thorpe Park (elevated mood, heightened energy), the celebrity Oxford rower Matthew Pinsent rowing and winning the medal in the 2004 Olympics (elevated and expansive mood, inflated self-esteem and grandiosity, goal attainment), Oxford undergraduate students exiting their final honours exam and being met by crowds of friends (elevated and expansive mood, inflated self-esteem and grandiosity, goal attainment), an Oxford University graduation ceremony (expansive mood, inflated self-esteem, goal attainment), people dancing at a club night (elevated mood, decreased need for sleep, heightened energy), Oxford ‘May Day’ celebrations involving people charging a police line and jumping off a bridge into a river (expansive mood, thrill seeking) and Tom Cruise publicly announcing his love for Katie Holmes (elevated and expansive mood, grandiosity). The idea of creating a positive film by drawing on mania-relevant material was inspired by Gruber et al. (2008), which investigated emotional and physiological responses to positive, negative and neutral film clips amongst people categorized as being at low or high risk for mania as measured by a self-report questionnaire (see also Gruber, Dutra, Eidelman, Johnson, & Harvey, 2011).

Verification of the relevance of the film clips to diagnostic features of mania was carried out by asking three clinicians (two psychiatrists and one clinical psychologist) working in a specialist mood disorders clinic to independently endorse whether each clip was relevant to each of the mania-related themes for which we had selected it. Agreement with the film construction in terms of mania-related themes as detailed above (e.g., elevated or expansive moody, grandiosity, inflated self-esteem) was high. The clinicians rated their endorsement/or non-endorsement for each separate theme per clip (*n* = 22). This yielded the following endorsement rates: 95.5%, 100% and 77.3%, giving a mean agreement of 90.9%.

#### Visuospatial task

As in previous studies (Holmes, James, et al., 2009; Holmes et al., 2010), the visuospatial task used was the computer game ‘Tetris’ (Green & Bavelier, 2003; Tetris Zone, 2007). The game involves using the cursor keys to move and rotate falling blocks to complete rows of blocks across the screen.

#### Intrusion diary

Participants were asked to keep a simple daily diary to record the occurrence (frequency) and content of involuntary memories, described as ‘intrusions’, from the positive film that they experienced over the 7 days following the experimental session. Intrusions were described as spontaneously occurring image-based involuntary memories from the positive film. The A5 diary was adapted from previous studies (Bourne et al., 2010; Holmes, James, et al., 2009; Holmes et al., 2004; Holmes et al., 2010) and structured so that each day was divided into a grid for *morning*, *afternoon* and *evening*. The intrusion frequency data (number of intrusions in that period) were entered into these grids. The corresponding content for each intrusion was recorded on lined pages also within the diary, specifically ‘Was it an image (I)?’ and ‘What was the content of the intrusion?’. Participants wrote a brief description of the involuntary memory in this space (e.g., ‘the rowers crossing the finishing line’). If participants had experienced no intrusions during any period, they were asked to write zero in the diary for that period. If an entry from the diary was not an image or could not be identified as being from the positive film, it was not included as an involuntary memory. Participants were asked to record all intrusions immediately after they occurred (whenever possible) and to set aside a regular time slot each day to check that their diary was up to date. This aimed to ensure that intrusions were not omitted if it had been impractical to record an intrusion immediately.

Participants were given instructions about the different forms intrusions can take: ‘What goes through our minds can either take the form of words and phrases (“verbal thoughts”), or it can be like mental images. Although mental images often take the form of pictures they can actually include any of the five senses, so you can imagine sounds or smells too. Mental images can be fuzzy or fragmented as well as clear; they can also be brief and very fleeting—like a quick flashback of the film.’ Participants were talked through an example of a mental image and an example of making an entry in the diary. In addition, while explaining the diary, the researcher checked comprehension and emphasized the following aspects of the spontaneous, image-based nature of involuntary memories from the film: that over the few days after the experimental session, participants may experience images of scenes from the film ‘popping’ into their mind when they were not expecting them; that mental images may be fuzzy, very brief or almost like a flash in your mind's eye; that even if participants experienced several intrusions that were the same to record each individual one in the diary every single time it occurred; and that we were interested in knowing about images but not about ‘verbal thoughts’ relating to the film.

#### Intrusion provocation task

Following previous studies (Krans, Naring, Holmes, & Becker, 2009; Lang, Moulds, & Holmes, 2009), an intrusion provocation task was used as a measure of the number of involuntary memories triggered by a visual reminder of the film. Participants viewed seven still pictures taken from the positive film. They were then required to sit with their eyes closed for 3 min and record any involuntary memories of the film experienced during this 3-min period.

### Baseline Questionnaire Measures

#### Beck Depression Inventory—Second Edition (Beck, Steer, & Brown, 1996)

The Beck Depression Inventory—Second Edition is a widely used self-report measure of depressive symptoms with robust reliability and validity (Beck et al., 1996).

#### Mood Disorder Questionnaire (Hirschfield et al., 2000)

The Mood Disorder Questionnaire has been used as a measure of bipolar risk in analogue studies of emotional processing (Rock, Goodwin, & Harmer, 2010a, 2010b). It comprises three primary questions. The first question contains 13 yes/no items indexing manic/hypomanic symptoms. The second question addresses the co-occurrence of symptoms, and the third question requires participants to report the severity of the symptoms on a four-point scale: no/minor/ moderate/serious problem. In the current study, the scoring reported by Calabrese et al. (2006) was used, in which seven out of all 15 questions renders a categorization as high risk to bipolar disorder.

#### State–Trait Anxiety Inventory (Spielberger, Gorsuch, Lushene, Vagg, & Jacobs, 1983)

Both the trait (State–Trait Anxiety Inventory-T) and state (State–Trait Anxiety Inventory-S) scales were used. The trait scale consists of 20 anxiety-related items for which participants rate ‘how you generally feel’, and the state scale consists of 20 anxiety-related items for which participants rate ‘how you feel right now, that is at this moment’. These are widely used measures and are reported to have satisfactory reliability and validity (Spielberger et al., 1983).

#### Spontaneous Use of Imagery Scale (Reisberg, Pearson, & Kosslyn, 2003)

The Spontaneous Use of Imagery Scale provides a measure of participants' everyday use of imagery. The questionnaire consists of 12 items, e.g., ‘When I think about a series of errands I must do, I visualise the stores I will visit’. Each item is rated on a five-point scale (1 = *never appropriate* and 5 = *always completely appropriate*).

### Questionnaire Measures Given at Baseline and Post-film

#### Positive and Negative Affect Schedules (Watson & Clark, 1994)

The 21 positive items from the expanded version of the Positive and Negative Affect Schedules (PANAS) were used to provide a measure of state positive affect, as in previous studies (e.g., Holmes, Mathews, Dalgleish, & Mackintosh, 2006). The positive subscales in the expanded PANAS comprise the basic positive emotion scales (joviality, eight items; self-assurance, six items; attentiveness, four items) as well as the serenity subscale (three items), as detailed by Watson and Clark (1994). Participants rate the 21 adjectives according to the extent to which they ‘feel this way now/in the past few minutes’ on a five-point scale (1 = *not at all* and 5 = *extremely*).

#### Willingly Approached Set of Statistically Unlikely Pursuits (Johnson & Carver, 2006)

The Willingly Approached Set of Statistically Unlikely Pursuits (WASSUP) provides a measure of the tendency to set extremely ambitious life goals that are mania-relevant. The original WASSUP consists of 30 extreme goals such as ‘someone will write a book about your life’ and requires participants to indicate how likely they are to ‘set the following goals for themselves’ on a five-point scale (1 = *no chance* and 5 = *definitely WILL*). We used a shortened version consisting of the 11 items that make up the ‘popular fame’ and ‘wealth’ subscales, following the studies by the authors of the scale that highlighted these subscales as most relevant to mania (Carver & Johnson, 2009; Johnson, Eisner, & Carver, 2009).

### Manipulation Checks Post-film

Participants marked 10-cm visual analogue scale ratings anchored from *not at all* to *extremely* for the statements: ‘Please indicate how much attention you have paid to the film you have just seen’, ‘Please indicate how much you enjoyed the film you have just seen’ and ‘Please indicate how personally relevant you found the film’ (scoring to nearest millimetre).

At 1 week, participants rated ‘How much do you predict that performing the Tetris task after a positive film (rather than watching it normally) would increase or decrease intrusive images of the film of the type you recorded in your diary?’ from −10 (*extreme decrease*) to 10 (*extreme increase*). Participants also rated how accurate they thought their completion of the diary to be from 1 (*not at all accurate*) to 10 (*extremely accurate*), and they rated the truth of the statement ‘I have often been unable (or forgotten) to record my intrusive images in the diary’ from 1 (*not at all true*) to 10 (*extremely true of me*).

Participants completed a Recognition Memory Task as a measure of voluntary memory for the positive film. Participants were presented with a questionnaire comprising a list of 14 written statements describing the film (e.g., ‘Only boats of 4 are shown’), to which they had to tick a box for ‘yes’ or ‘no’. The written statements were anchored by titles referring to the seven scenes from the film (e.g., ‘Scene 2: Rowing Montage’), with two statements per scene.

### Procedure

Participants provided their written informed consent and completed baseline assessments of depression, history of hypomanic symptoms, use of mental imagery, state and trait anxiety, state positive mood and goal setting. Participants then viewed the positive film. To promote self-involvement, participants were instructed to imagine that they were the main character in each scene of the film and watched the film alone in a darkened room. Participants then repeated measures of state positive mood and goal setting and manipulation check ratings. They were then randomly assigned to one of the two conditions (visuospatial task versus no task). Participants in the visuospatial task condition played Tetris for 10 min, whereas those in the no-task condition were asked to sit quietly for 10 min.

Participants kept the intrusion diary over 1 week, at the end of which they returned to the research centre. At this follow-up session, the researcher went through the diary to verify that the content of each of the involuntary memories came from a scene in the positive film. Participants then completed the Demand and Compliance ratings, Recognition Memory Test and Intrusion Provocation Task. Finally, participants were debriefed.

## RESULTS

### Statistical Analyses

Baseline characteristics of participants in the visuospatial and no-task conditions were compared using two-tailed independent-samples *t* tests or chi-squared tests of independence. Two-tailed independent-samples *t* tests were used to compare the differences in post-film and follow-up measures between the visuospatial and no-task conditions. A mixed ANOVA with a within-subjects factor of time (pre-film versus post-film) and a between-subjects factor of condition (visuospatial versus no task) was used to investigate changes over the course of the film. An alpha level of .05 was used for all statistical tests.

### Participant Characteristics at Baseline

Participants' characteristics at baseline were compared between the two conditions (visuospatial versus no task); see Table for details. This revealed no significant differences between groups on demographic or questionnaire measures, suggesting successful randomization.

**Table:** Participant characteristics at baseline (pre-film) by condition

	Visuospatial (*n* = 36)	No task (*n* = 33)	*t*(67)	*p*
*M* (*SD*)	*M* (*SD*)
Age (years)	20.33 (0.89)	20.30 (0.84)	0.14	.89
Gender (% female)	58	51	*χ*^2^ = .32	.57
Ethnicity (% Caucasian)	89	88	*χ*^2^ = .02	.90
BDI-II	7.64 (5.63)	9.48 (7.68)	1.15	.26
MDQ	7.89 (4.58)	7.91 (4.30)	0.19	.99
STAI-T	39.11 (7.69)	41.36 (11.96)	0.92	.36
STAI-S	32.97 (6.41)	35.10 (9.99)	1.06	.29
SUIS	36.39 (8.73)	39.42 (7.41)	1.55	.13
PANAS (pre-film)	59.50 (13.79)	58.00 (11.14)	0.49	.62
WASSUP (pre-film)	18.78 (6.53)	21.09 (8.21)	1.30	.20

BDI-II = Beck Depression Inventory—Second Edition; MDQ = Mood Disorders Questionnaire; STAI-T/S = State–Trait Anxiety Inventory, trait/state version; SUIS = Subjective Use of Imagery Scale; PANAS = Positive Scale of Positive and Negative Affect Schedules, state version; WASSUP = Willingly Approached Set of Statistically Unlikely Pursuits.

### Immediate Effects of Positive Film

PANAS and WASSUP scores were analysed using a mixed-model ANOVA with a within-subjects factor of time (pre-film versus post-film) and a between-subjects factor of condition (visuospatial versus no task); for mean scores, see Tables 1 and 2. For the PANAS, as predicted, there was a significant main effect of time, *F*(1, 67) = 24.19, *p* < .001, *η*² = .27,^1^ and no significant interaction of time with condition, *F*(1, 67) < 1, indicating that both groups experienced a similar increase in positive affect over the course of watching the film. For the WASSUP, as predicted, there was a significant main effect of time, *F*(1, 67) = 4.35, *p* = .04, *η*² = .061, and no significant interaction of time with condition, *F*(1, 67) < 1, indicating that both groups experienced an increase in willingness to set ambitious goals over the course of watching the film.

**Table:** Measures of mood, goal setting, involuntary memories and manipulation checks immediately after the positive film and at 1-week follow-up

	Visuospatial (*n* = 36)	No task (*n* = 33)		
*M* (*SD*)	*M* (*SD*)	*t* (67)	*p*
PANAS				
Post-film	66.58 (15.19)	63.91 (14.64)	0.74	.46
WASSUP				
Post-film	19.47 (7.34)	21.76 (8.89)	1.17	.25
Number of involuntary memories				
Diary	1.44 (1.65)	4.00 (5.69)	2.58	.01
Provocation task	6.89 (7.46)	9.12 (9.60)	1.08	.28
Film ratings				
Attention	8.33 (1.74)	8.53 (0.91)	0.58	.56
Enjoyment	6.89 (1.80)	6.97 (1.64)	0.19	.85
Personal relevance	6.37 (2.23)	6.45 (1.86)	0.16	.87
Diary ratings				
Compliance	2.31 (1.85)	2.24 (1.75)	0.15	.89
Accuracy	8.44 (1.56)	8.24 (1.44)	0.56	.58
Expectancy	−2.28 (4.43)	−1.09 (3.45)	1.23	.22
Recognition memory	9.25 (1.38)	9.97 (1.40)	2.15	.04

PANAS = Positive Scale of Positive and Negative Affect Schedules, state version; WASSUP = Willingly Approached Set of Statistically Unlikely Pursuits; Recognition memory = score on recognition memory test.

### Effects of Experimental Condition on Involuntary Memories

All intrusions recorded in the 7-day diary related directly to the film. Participants in the visuospatial condition recorded significantly fewer involuntary memories of the film in the 7-day diary than participants in the no-task condition, *t*(67) = 2.58, *p* = .012, *d* = 0.62 (Figure 1). There was no significant difference between the two conditions on number of involuntary memories following the intrusion provocation task, *t*(67) = 1.08, *p* = .28.

**Figure:**
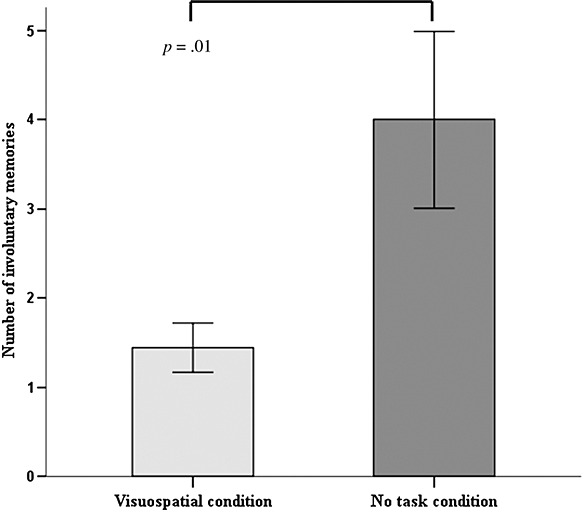
Mean number of involuntary memories of scenes from the positive film over 1 week. Standard errors are represented in the figure by the errors bars attached to each column

### Manipulation Checks

#### Experience of the positive film

There were no significant differences between the two conditions on ratings of attention paid to the film, enjoyment of the film or personal relevance (all *t*s < 1, Table 2).

#### Diary compliance and demand

There were no significant differences between the two conditions on ratings of diary compliance or accuracy (*t*s < 1) or demand expectations, *t*(67) = 1.23, *p* = .22 (Table 2).

#### Recognition memory test

Participants in the visuospatial task condition scored significantly lower on the recognition memory test than participants in the no-task condition, *t*(67) = 2.15, *p* = .04, *d* = 0.52. As a *post hoc* analysis to check that this difference in recognition memory did not account for differences between the two groups on number of involuntary memories, an analysis of covariance was conducted with between-subjects factor of condition (visuospatial versus no task) and with recognition memory test score as covariate. The between-subjects effect of condition remained significant (*F*(1, 66) = 5.07, *p* = .028, *η*² = .071), whereas the effect of the covariate was non-significant (*F*(1, 66) < 1), suggesting that the difference in number of involuntary memories recorded between the two conditions was not merely an effect of differences in voluntary recognition memory.

## DISCUSSION

The current study investigated the development of positive image-based involuntary memories after viewing a ‘positive film’. The ‘positive’ themes were generated by considering diagnostic criteria for mania and developing the film to be specifically relevant to the study population in Oxford. Thus, this paradigm relates to the ‘trauma film’ paradigm hitherto used to study negative involuntary memories (Holmes & Bourne, 2008), adapted to allow the study of positive involuntary memories. Our first findings concern the development of this experimental paradigm. Viewing the positive film enhanced positive mood and also increased ambitious goal setting on a questionnaire measure (the WASSUP; Johnson & Carver, 2006) immediately post-film. The positive film was also successful in generating positive involuntary memories of specific scenes over the next 7 days. This is the first study to our knowledge to generate positive involuntary memories in a controlled laboratory setting. This study demonstrates that a ‘positive film’ paradigm can be used to investigate the formation and the occurrence of spontaneous positive imagery.

Our second key findings concern the experimental manipulation. As predicted, compared with those allocated to a no-task control condition, participants who engaged in a visuospatial task (Tetris) immediately after watching the positive film experienced significantly fewer positive involuntary memories from the film over the next week. In addition, participants in the visuospatial condition showed impaired voluntary recall of the film at 1 week, as indicated by scores on a recognition memory task. This suggests that although involuntary memory and voluntary memory can be independent (Bourne et al., 2010), in this study, the task dampened both types of recall. Overall, this study provides some preliminary evidence that factors that can dampen down the formation of negative involuntary memories (Holmes, James, et al., 2009; Holmes et al., 2010) can also have the same impact on positive involuntary memories.

Are there potential clinical implications regarding the modulation of positive imagery after a positive emotional experience? First, let us consider a clinical disorder that may include an excess of spontaneous positive imagery—(hypo)mania in bipolar disorder (Gregory et al., 2010; Holmes, Geddes, et al., 2008). The positive film produced a potential analogue of certain mania-related experiences, i.e., spontaneous positive images. Thus, it may be possible to use this paradigm in developing our knowledge of the underlying mechanisms of such experiences in abnormally elevated mood states. The success of this paradigm may be particularly encouraging as some other non-imagery-based mood induction procedures have struggled to produce mania-related effects (e.g., Clark, Iversen, & Goodwin, 2001). In terms of future clinical translation, there may be a role for simple visuospatial tasks in dampening down the impact of spontaneous positive imagery in fuelling mania in a similar way as has been proposed for dampening down flashbacks in PTSD (Holmes, James, et al., 2009; Holmes et al., 2010; Lilley, Andrade, Turpin, Sabin-Farrell, & Holmes, 2009). It may be that such an approach could even be extended to other areas where spontaneous ‘positive’ imagery can be problematic such as suicidality (Crane et al., 2012; Hales et al., 2011; Holmes et al., 2007) and cravings (May et al., 2004).

Second, people with depression suffer from a deficit in positive mental imagery (e.g., Holmes, Lang, et al., 2008; Joormann & Siemer, 2004; Werner-Seidler & Moulds, 2011) and a proliferation of negative mental imagery (e.g., Patel et al., 2007; Starr & Moulds, 2006; Williams & Moulds, 2007). In terms of future clinical translation, the investigation of spontaneous positive imagery using the positive-film paradigm may highlight ways of enhancing interventions, both for depression and for other disorders, in which deficits in positive imagery play a key role (e.g., Arntz & Weertman, 1999; Blackwell & Holmes, 2010; Mooney & Padesky, 2000). Imagery might be boosted through use of positive films as well as by positive internally generated imagery (Holmes, Lang, & Shah, 2009). In fact, it is possible that some of the beneficial effects of deliberately generated positive imagery may occur via this imagery returning in spontaneous form later on. In a recent study, people with depression engaged in a computer task that involved repeatedly having to generate positive imagery in response to auditory descriptions of everyday situations (Blackwell & Holmes, 2010). Some participants spontaneously reported experiencing involuntary memories of the training scenarios in their daily lives, which they felt had an impact on their mood and sometimes even their behaviour. These preliminary findings require continued formal investigation (e.g., Lang et al., 2012). However, these novel approaches that aim to boost positive imagery in depression are an exciting area of development. The current study suggests that after a positive emotional experience, engaging in a visuospatial task (even inadvertently in daily life, such as playing visual computer games) may reduce the likelihood of subsequently experiencing the very type of spontaneous positive mental images that may promote positive mood and helpful goal-directed behaviour.

The study has a number of limitations. The study population was students with a restricted age range, which limits the generalizability of the findings. Additionally, although the number of involuntary memories was monitored, their impact on emotion and behaviour was not recorded in the diary. This will be important to address in future studies. Any difference in involuntary memories between the two conditions on the intrusions provocation test did not reach significance. Following the method used in the initial study demonstrating the effect of Tetris in reducing involuntary memories from the trauma film (Holmes, James, et al., 2009), the current study used a ‘no-task’ rather than active control condition, and thus, we cannot conclude from this study that the effects of playing Tetris on involuntary memories from the positive film were specifically due to the visuospatial nature of the task, rather than non-specific effects such as distraction, or due to cognitive processes such as rumination increasing involuntary memory formation in the control group. Although the specificity of the visuospatial Tetris task for reducing involuntary memories of negatively valenced material (the trauma film) has been demonstrated, e.g., in comparison with the verbal ‘Pub Quiz’ game (Holmes et al., 2010), whether this specificity also applies to involuntary memories of positively valenced material, such as from the positive film, is an important question to address in future studies. Finally, further research is needed on the rates of spontaneous positive imagery in different populations.

A better understanding of positive cognition, e.g., in the neglected area of spontaneous positive images, may yield insights to enhance well-being and improve mental health. Moreover, given the ubiquity of spontaneous thoughts, both positive and negative, in our daily lives and their potential impact on our mood and behaviour (Berntsen, 1996; Berntsen & Jacobsen, 2008; D'Argembeau et al., 2011; Pillemer, 1988), the study raises intriguing questions as to the potential impact of everyday tasks on such experiences. If the likelihood of an event returning as a later ‘flashback’, whether positive or negative, can be affected significantly by simple laboratory tasks, what is the impact on our experiences of spontaneous memories or future-oriented thoughts of the kinds of tasks that we engage in during everyday life?

## Key Practitioner Message

- After a positive film, volunteers can experience involuntary ‘pop-out’ memories of that film over the subsequent week.
- Performing a visuospatial task (the computer game Tetris) immediately after the positive film reduces spontaneous image memories and also impairs voluntary memory for details of the film.
- Understanding the mechanisms underlying positive spontaneous imagery may offer insights for treatment innovation both for those conditions characterized by a deficit in positive imagery, such as depression, and for those characterized by a proliferation of positive imagery, such as mania.
- Furthermore, this may suggest that the routine everyday tasks we engage in after a positive emotional event could have a significant impact on our memories of positive experiences.

## Footnotes

1 Effect sizes given by partial *η*² are *η*² = .01 for small, *η*² = .059 for medium and *η*² = .138 for large effect sizes (Clark-Carter, 2010).
